# Acute and Chronic Effects of High Frequency Electric Pulse Stimulation on the Akt/mTOR Pathway in Human Primary Myotubes

**DOI:** 10.3389/fbioe.2020.565679

**Published:** 2020-11-05

**Authors:** Mayalen Valero-Breton, Geoffrey Warnier, Mauricio Castro-Sepulveda, Louise Deldicque, Hermann Zbinden-Foncea

**Affiliations:** ^1^Exercise Physiology Laboratory, School of Kinesiology, Universidad Finis Terrae, Santiago, Chile; ^2^Institute of Neuroscience, UCLouvain, Louvain-la-Neuve, Belgium; ^3^Centro de Salud Deportiva, Clínica Santa María, Santiago, Chile

**Keywords:** Akt, mTOR, ERK1/2, high frequency, cell culture

## Abstract

Electrical pulse stimulation (EPS) has been suggested to be a useful method to investigate the mechanisms underlying the adaptations of human skeletal muscle to both endurance and resistance exercise. Although different myotube stimulation protocols mimicking acute and chronic endurance exercise have been developed, no convincing protocol mimicking resistance exercise exists. Adaptations to resistance exercise mainly ensue via the Akt/mTOR pathway. Therefore, the aim of this study was to develop a high frequency EPS protocol mimicking resistance exercise both acutely (100 Hz, 15 V, 0.4 ms with 4 s rest between each contraction for 30 min) and chronically (acute EPS protocol repeated on three consecutive days) on human myotubes. Compared to control conditions, the acute EPS protocol increased the phosphorylation of Akt^Ser473^ at 0 h (+91%, *p* = 0.02) and 3 h (+95%, *p* = 0.01), and mTOR^Ser2448^ at 0 h (+93%, *p* = 0.03), 1 h (+129%, *p* = 0.01), and 3 h (+104%, *p* = 0.0250) post-stimulation. The phosphorylation of ERK1/2^Thr202/Tyr204^ was increased at 0 h (+69%, *p* = 0.02) and 3 h (+117%, *p* = 0.003) post-stimulation compared to control conditions. In addition, both S6K1^Thr389^ (+157%, *p* = 0.009) and S6^Ser240/244^ (+153%, *p* = 0.003) phosphorylation increased 1 h after EPS compared to control conditions. Chronic EPS protocol increased the phosphorylation of S6K1^Thr389^ 1 h (+105%, *p* = 0.03) and 3 h (+126%, *p* = 0.02) and the phosphorylation of S6^Ser240/244^ 1 h (+32%, *p* = 0.02) after the end of the last stimulation. In conclusion, the present work shows that human muscle cells subjected to EPS can be used as an *in vitro* model of acute and chronic resistance exercise.

## Introduction

Electrical pulse stimulation (EPS) of muscle cells is an *in vitro* exercise model that mimics muscle adaptations *in vivo* (Manabe et al., 2012; Carter and Solomon, 2019). Motor unit activation can be replaced by subjecting cultured human myotubes to EPS (Nikolić and Aas, 2019). EPS has been suggested to be a useful method to investigate the mechanisms underlying acute and chronic adaptations of human skeletal muscle to both endurance (Lambernd et al., 2012) and resistance exercise (Görgens et al., 2015; Tarum et al., 2017). Generally, resistance exercise induces a greater recruitment of muscle fibers than endurance exercise, which in turn translates into a higher frequency of neuromuscular firing (De Luca et al., 2014). To date, several studies have shown low frequency EPS (0.2–2 Hz) to act as an endurance exercise mimetic (Lambernd et al., 2012; Nikolić et al., 2012; Brown et al., 2015). Similarly, medium frequency EPS (1–30 Hz) has been suggested to act as a resistance exercise mimetic in human muscle cells and C2C12 cells (Scheler et al., 2013; Tarum et al., 2017). A study in human has shown that 100 Hz allowed ∼70% of the maximal voluntary contraction to be produced (Dreibati et al., 2010), however, it is unknown whether high frequency protocols induce typical resistance exercise adaptations in human muscle cells. As stimulation at either high or low frequencies appear to activate different signaling pathways (Nikolić et al., 2012), it is crucial to carefully investigate the adaptations to high frequency stimulation (100 Hz).

Resistance exercise increases both muscle strength and size, primarily by stimulating protein synthesis, which is itself under the control of the Akt/mammalian target of rapamycin (mTOR) pathway (Hughes et al., 2018). Activation of mTOR results in the phosphorylation of S6 kinase 1 (S6K1) and its downstream target the ribosomal protein S6 (rpS6) (Ma and Blenis, 2009; Marabita et al., 2016). In addition, the MAPK pathway, or more specifically ERK1/2, also contributes to the adaptations occurring in skeletal muscle in response to resistance exercise (Moore et al., 2011). In this sense, neuromuscular electrical stimulation at 60 but not 30 Hz increased the phosphorylation of mTOR and S6K1 in human skeletal muscle (Mettler et al., 2018). Similar results were obtained in isolated rat muscle when comparing 100 and 10 Hz stimulation, only the highest frequency activated the mTOR pathway (Atherton et al., 2005). Accordingly, the investigators surmised that high-frequency stimulation resulted in a “hypertrophy-like” adaptation similar to that achieved by resistance training.

To date, most studies involving EPS have been conducted in the murine cell line C2C12 or in primary skeletal muscle cells of animal origin (Nikolić et al., 2017). The advantage of human primary myotubes is that they have greater sarcomere contractile activity than C2C12 myotubes (Manabe and Fujii, 2016; Manabe et al., 2016), and also preserve phenotypical characteristics of the donor. Therefore, the aim of the present study was to develop an acute and chronic EPS protocol in human primary myotubes to 100 Hz mimicking resistance exercise *in vivo* and validate this by assessing the Akt/mTOR pathway activation.

## Materials and Methods

### Muscle Tissue Explants

Human muscle biopsies of the vastus lateralis muscle were obtained from young, healthy, and sedentary men (*n* = 3) as previously described (Gnimassou et al., 2018). Immediately after collection, a fraction of tissue of approximately 20–30 mg was washed in sterile PBS and minced into small segments. Segments were placed in a 35-mm plate coated with Matrigel^®^ (6 mg/ml, BD Biosciences) and maintained in growth media [DMEM (Sigma-Aldrich), 20%v/v fetal bovine serum (FBS) (GIBCO, Thermo Fisher Scientific), and 0.5%v/v Ultroser^TM^ G (Pall corporation, United States)] in a cell incubator at 37°C in 95% humidified air, with 5% CO_2_. Plates were incubated for 5–7 days. During this time, primary cells migrated out of the explant and monitored daily via a microscope up to covering the plate surface. Subsequently, the outgrown cells were cultured until a confluent cell monolayer was obtained. Cells were enzymatically harvested using dispase (BD Biosciences), sub-cultured in growth medium, and then sorted using magnetic activated cells sorting (MACS©, Miltenyi Biotec, Germany) with magnetic microbeads directly linked to an antibody against the specific cell surface marker CD56 (purified mouse, anti-human CD56, BD-Biosciences, cat# 559043). The study was approved by the ethical committee of Saint-Luc Hospital/UCLouvain (Belgium). The safety procedures of the UCLouvain were applied whenever needed. The study was performed according to the Declaration of Helsinki.

### Cell Proliferation and Differentiation

For both acute and chronic experiments, 1 × 10^5^ myoblasts were seeded in 35-mm diameter culture dishes in 6-well plates and incubated at 37°C in a humidified air atmosphere with 5% CO_2_. The cells were counted with a cell counting equipment (BLAUBRAND^®^, counting chamber, Brand, Germany). The primary culture protocol was the same for the control and EPS conditions in which the cells were grown in DMEM (Life Technologies) supplemented with 20% FBS, 1% penicillin/streptomycin, 0.5% Ultroser G. When cells were 70% confluent, the proliferation medium was replaced by a differentiation medium (DMEM) containing 2% FBS and 1% penicillin/streptomycin. The differentiation medium was replaced every 2 days and on differentiation day 7, fully mature myotubes were stimulated with EPS. In this case, the medium was replaced prior to the stimulation in both the control and stimulated conditions.

### Electric Pulse Stimulation

On differentiation day 7, differentiated primary myotubes were electrically stimulated using the C-Pace EP Cell culture stimulator, with C-dish and carbon electrodes (IonOptix, Milton, MA, United States) in stimulation medium (DMEM, low glucose media supplemented with 1% serum) while the control group remained with the same stimulation medium but without the carbon electrodes. EPS experiments were performed in parallel plates with cells seeded at the same time and same cell culturing conditions. The acute protocol consisted of a single stimulation at 100 Hz, 15 V, 0.4 ms with 4 s rest between each contraction for 30 min (day 7 of differentiation). The chronic protocol consisted of repeating the acute protocol over three consecutive days, at the same time every day, from day 7 to day 9 of differentiation.

In both protocols, once the stimulation period was completed, the plates were washed with cold PBS and cell lysis performed 0, 1, and 3 h after the intervention.

### Protein Extraction

Cells were rinsed once with PBS and harvested in an ice-cold lysis buffer containing 20 mM Tris, pH 7.0, 270 mM sucrose, 5 mM EGTA, 1 mM EDTA, 1% Triton X-100, 1 mM sodium orthovanadate, 50 mM sodium β-glycerophosphate, 5 mM sodium pyrophosphate, 50 mM sodium fluoride, 1 mM DTT (1,4 dithiothreitol), and a protease inhibitor cocktail containing 1 mM EDTA (Roche Applied Science, Belgium). The homogenates were then centrifuged at 10,000 *g* for 10 min at 4°C. The supernatants were immediately stored at −80°C. Protein concentration was determined using the DC protein assay kit (Bio-Rad Laboratories, Nazareth, Belgium).

### Western Blot Analyses

Cell lysates (15 μg) were combined with Laemmli sample buffer and warmed for 5 min at 95°C before being loaded on gels. Samples were separated by SDS/PAGE (10%). After electrophoretic separation at 40 mA for 1 h, the proteins were transferred to PVDF membranes at 80 V for 3 h for western blot analysis. Membranes were blocked for 60 min in Tris-buffered saline with 0.1% Tween 20 (TBST) and 5% milk. Subsequently, membranes were incubated with the following antibodies (1:1000) overnight at 4°C: phospho-AktSer473, phospo-mTOR Ser2448, phospho-S6K1 Thr389, phospho-S6 ribosomal Ser240/244, phospho-4E-BP1 Thr37/46, and phospho-ERK Thr202/Tyr204. Antibodies (obtained from Cell Signaling) were diluted in TBST containing 1% BSA. Membranes were washed three times in TBST and incubated with a secondary antibody at room temperature for 60 min [anti-rabbit (1:10000) or anti-mouse (1:10000) from Sigma (Bornem, Belgium)]. After an additional three washes, chemiluminescence detection was carried out using the ECL western Bright quantum HRP substrate (Isogen). The films were scanned on GeneSnap software via the G-box iChemi XL machine (Syngene, Cambridge, United Kingdom) and quantified with ImageJ software (NIH, Bethesda, MD, United States). The results represent the phosphorylated form of the protein. Phosphorylation ratios were calculated by dividing the phosphorylation levels by red Ponceau staining. A value of one was arbitrarily assigned for each control to which the respective stimulated condition was reported, to compare the basal state of our participants.

### Statistical Analyses

The EPS and control groups were compared using an unpaired Student’s *t-*test. Results are shown as means ± SEM. The data obtained were analyzed statistically using the GraphPad Prism 6 program (United States). A *p* < 0.05 was considered significant.

## Results

### Acute Protocol

**FIGURE 1:**
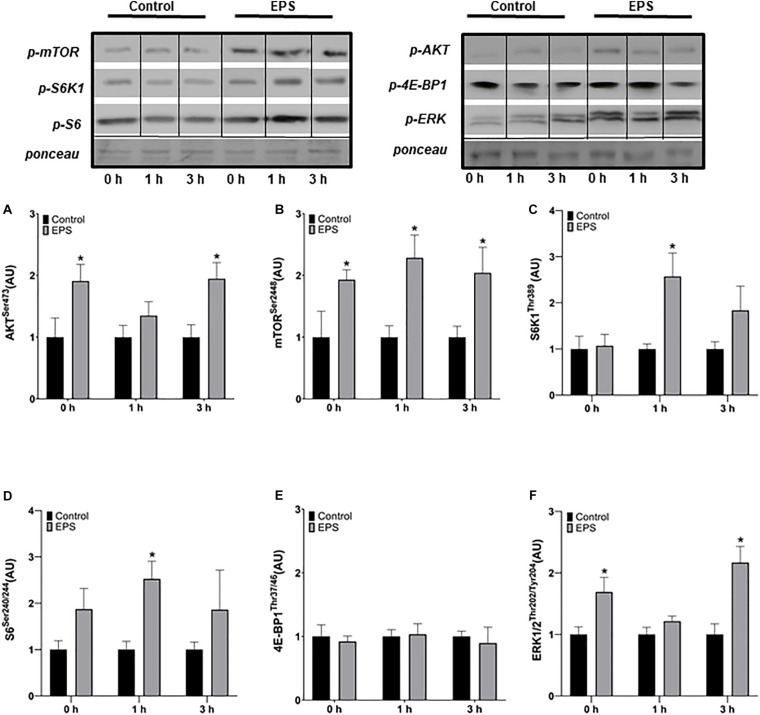
Phosphorylation of Akt^Ser473^ **(A)**, mTOR^Ser2448^ **(B)**, S6K1^Thr389^ **(C)**, S6^Ser240/244^ **(D)**, 4E-BP1^Thr37/46^ **(E)**, and ERK1/2^Thr202/Tyr204^ **(F)** after an acute bout of electric pulse stimulation. *Significantly different from the control group after the intervention (*p* < 0.05). The phosphorylation ratios were calculated by dividing the phosphorylation levels by Ponceau staining. The values in the graphs correspond to the means ± SEM.

The phosphorylation of Akt was increased at 0 h (+91%, *p* = 0.0283) and 3 h after stimulation (+95%, *p* = 0.0103) in EPS compared to control conditions, but not at 1 h (Figure 1A). The phosphorylation of mTOR increased at 0 h (+93%, *p* = 0.0364), 1 h (+129%, *p* = 0.0071), and 3 h (+104%, *p* = 0.0250) after EPS compared to control conditions (Figure 1B). The phosphorylation of S6K1 (+157%, *p* = 0.0082; Figure 1C) and S6 (+153%, *p* = 0.0032; Figure 1D) increased 1 h after EPS compared to control conditions. No difference in 4E-BP1 phosphorylation was detected between EPS and control conditions at any time point (Figure 1E). Similarly to Akt, the phosphorylation of ERK1/2 increased at 0 h (+69%, *p* = 0.0163) and 3 h (+117%, *p* = 0.0028) after EPS compared to control conditions, but not at 1 h (Figure 1F).

### Chronic Protocol

**FIGURE 2:**
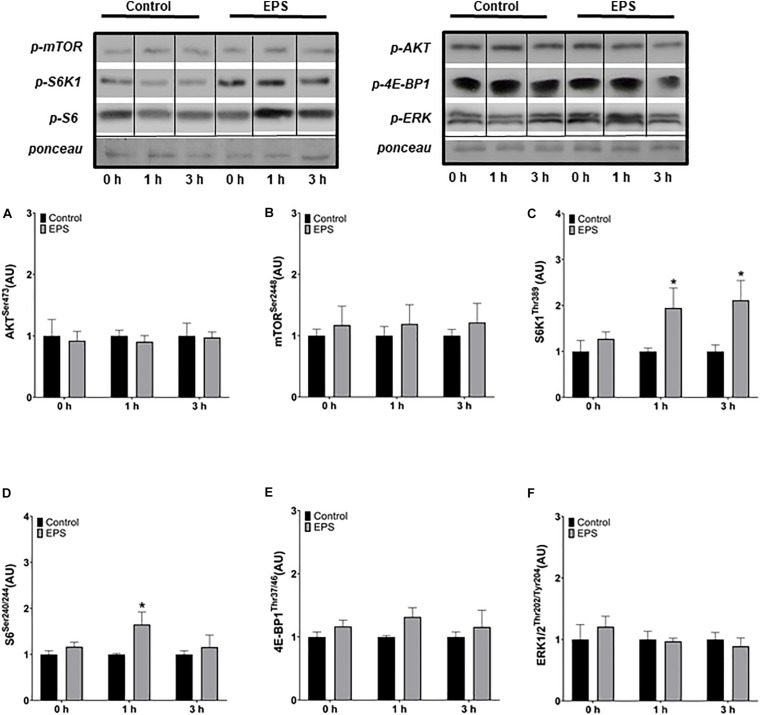
Phosphorylation of Akt^Ser473^ **(A)**, mTOR^Ser2448^ **(B)**, S6K1^Thr389^ **(C)**, S6^Ser240/244^ **(D)**, 4E-BP1^Thr37/46^ **(E)**, and ERK1/2^Thr202/Tyr204^ **(F)** after the last of three daily electric pulse stimulations. *Significantly different from the control group after the intervention (*p* < 0.05). The phosphorylation ratios were calculated by dividing the phosphorylation levels by Ponceau staining. The values in the graphs correspond to the means ± SEM.

No changes in Akt (Figure 2A) nor in mTOR (Figure 2B) phosphorylation was observed at any time point. However, the phosphorylation of S6K1 increased at 1 h (+105%, *p* = 0.0319; Figure 2C) and 3 h (+126%, *p* = 0.0196) and the phosphorylation of S6 at 1 h (+32%, *p* = 0.0195; Figure 2D) after the final of the three daily stimulations compared to control conditions. No changes in 4E-BP1 (Figure 2E) nor in ERK1/2 phosphorylation (Figure 2F) were observed following the chronic stimulation protocol.

## Discussion

The goal of the present work was to develop a high-frequency EPS protocol to mimic resistance exercise, and to validate this by showing an activation of the Akt/mTOR pathway in primary human myotubes. The main finding is that our protocol is indeed efficient at activating both the Akt/mTOR pathway in an acute setting and proteins downstream of mTOR such as S6K1 and S6 in a chronic setting.

Therefore, our results suggest that primary myotubes not only respond to acute high EPS frequency, but also adapt to chronic stimulation in a similar manner to resistance training.

Studies have shown an increase in the expression of slow-type fiber markers with low frequency stimulation at 2.5 Hz over a 2 week period (Wehrle et al., 1994) and at 1 Hz after 24 h of EPS compared to unstimulated cells (Nikolić et al., 2012). From this time, different EPS protocols were developed to selectively mimic endurance and resistance exercise as the adaptations that occur in human skeletal muscle in response to these two exercise modes depend on the selective activation of molecular sensors (Atherton et al., 2005). The AMP-activated protein kinase (AMPK) is mainly activated by endurance exercise whilst mTOR is mainly activated by resistance exercise (Hughes et al., 2018). Low frequency EPS (1 Hz) increases AMPK activity after acute, as well as after chronic stimulation (Nikolić et al., 2012), whilst EPS at a higher frequency (30 Hz) over 4 h increases the phosphorylation of S6K1 in human myotubes (Scheler et al., 2013). Whether other components of the Akt/mTOR pathway were activated by 30 Hz for 4 h was not tested. The parameters of the latter study seem to be more representative of high-intensity endurance exercise than resistance exercise, if any comparison with actual human exercise protocols can be made. Indeed, a 4-h stimulation does not mimic a session of resistance exercise in humans, which generally lasts between 30 and 90 min.

To better mimic the conditions in sports and rehabilitation, we stimulated our cells for 30 min whether acutely or repeatedly for 3 days, with a long recovery period between sessions. This protocol allowed to use a relatively high frequency of 100 Hz, which was found to increase protein synthesis in differentiated C2C12 cells (Donnelly et al., 2009) and in isolated rat muscle, concomitantly with the activation of the Akt/mTOR pathway (Atherton et al., 2005). Although we did not directly assess muscle protein synthesis, we did detect an activation of the Akt/mTOR pathway as well as increased ERK1/2 phosphorylation levels, at least acutely.

Repeating this protocol three times on three consecutive days decreased the sensitivity of the Akt/mTOR pathway to high-frequency stimulation. This is not surprising as resistance training has been shown to dampen the acute activation of protein synthesis and the mTOR pathway in skeletal muscle measured after a single session (Tang et al., 2008). The reduced activation of the mTOR pathway after our chronic protocol argues for a valid simulation of resistance exercise in our conditions *in vitro*. However, to the best of our knowledge, no study investigated the effect of only three consecutive sessions on the activation of the mTOR pathway in human skeletal muscle. Therefore, while plausible, this interpretation must be taken with caution.

The major limitation of the present study is that only markers for protein synthesis and muscle anabolism were measured. There was no direct assessment of muscle protein synthesis, nor of myotube diameter. The strength of our study is to have developed a model in primary human myotubes. Working on cells derived from human muscle biopsies is better than working on cell lines, as primary cells maintain the genetic and -to a certain extent- epigenetic background of the donor. This opens the perspective of being able to investigate the effect of contractile activity in a series of diseases and to understand the underlying molecular adaptations (Aas et al., 2013). This step is critical in the development of so-called exercise mimetics, i.e., drugs that induce similar beneficial effects as physical activity without, or only very limited, side effects, designed for people who cannot exercise or maintain the motivation to do it regularly.

In conclusion, the present work shows that human muscle cells subjected to EPS can be used as an *in vitro* model of acute and chronic resistance exercise. The parameters of stimulation developed here could be useful for future studies intending to investigate the molecular responses of acute and chronic resistance exercise in an *in vitro* model in the quest to develop exercise-mimetics.

## Data Availability Statement

All datasets presented in this study are included in the article/supplementary material.

## Author Contributions

MV-B, MC-S, LD, and HZ-F: study conceptualization and experimental design, manuscript writing, review and editing. MV-B and GW performed the experiments and data collection. All authors contributed to the article and approved the submitted version.

## Conflict of Interest

The authors declare that the research was conducted in the absence of any commercial or financial relationships that could be construed as a potential conflict of interest.
